# Case report series: revisiting third and fourth pharyngeal arch anomalies, − are they thymopharyngeal duct remnants?

**DOI:** 10.1186/s40463-020-00475-w

**Published:** 2020-12-11

**Authors:** A. O. Kotwica, J. Rudd, D. J. Howard

**Affiliations:** 1 Rhinology and Laryngology Research Fund Fellow, London, UK; 2 Great Ormond Street Hospital, London, UK; 3 Royal National Throat, Nose and Ear Hospital, London, UK

**Keywords:** Branchial arch, Pharyngeal arch, Neck abscess, Thymopharyngeal duct remnant

## Abstract

**Background:**

Pharyngeal arch anomalies are the second most common form of head and neck congenital defect. The second arch anomalies are the most common, and compromise 95% of cases. Little is known about the 3rd and 4th arch anomalies as they are extremely rare. They most commonly present in childhood with sudden severe left lateral neck infection and abscess formation with considerable tendency to recur, contributing to significant mortality and morbidity in those patients.

**Case presentation:**

Here we present four cases finally diagnosed as third or fourth pharyngeal arch anomalies, with more than 20 years of follow-up following their definitive surgery. The possibility that they are thymopharyngeal duct remnants is discussed.

**Conclusion:**

Meticulous open radical surgical excision of all involved paralaryngeal, parapharyngeal and thyroid tissue, with preservation of the superior and recurrent laryngeal nerves, is required for cure of recurrent cases.

**Graphical abstract:**

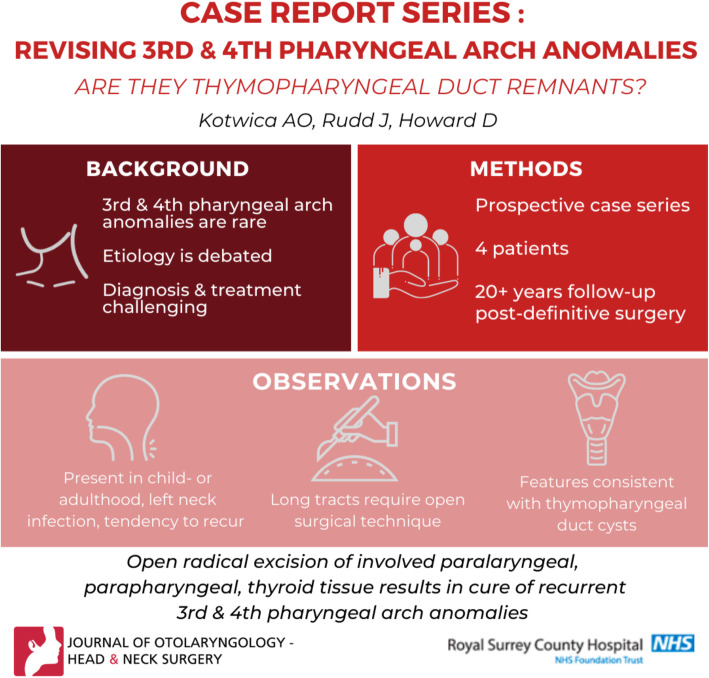

## Introduction

### Clinical aspects

Pharyngeal arch anomalies are the second most common form of head and neck congenital defect. In most reported series 2nd arch anomalies comprise approximately 95% of cases and 1st arch anomalies 3–4% [1]. The 3rd and 4th arch anomalies described in the medical and surgical literature are extremely rare. They most commonly present with sudden severe lateral neck infection and abscess formation, which may involve the thyroid gland. They are more common in children but can present primarily in adults [1]. The literature contains conflicting reports on the etiological basis of these lesions. Only a few centers in the world have any experience of multiple cases in children and adults. The accurate diagnosis and treatment remain a challenging subject. Endoscopic treatment has been advocated but lacks long-term follow-up studies. Here, we present the details of four cases, which required open definitive surgery by the senior author and have had follow-up for over 20 years. This case series may be particularly relevant to head and neck surgeons as well as paediatric otolaryngologists.

### Embryology

The cranial region of the early human embryo during the third week of development resembles more primitive forms of life, such as a fish embryo at the same stage. The study of the branchial apparatus by embryologists in other life forms has been, and indeed remains, essential for our understanding of its function. The term branchial (the Greek term “branchia” means gill) remains in common usage. However, humans do not develop gills and many authors now prefer the term pharyngeal arch.

The embryonic period of human development continues up to 8 weeks. Between the fourth and eight weeks the ancestral branchial apparatus becomes totally rearranged and adapted to new structures and functions, or alternatively disappears. It is during this complex transformation period that most congenital malformations occur. One third of all human congenital malformations occur in the head and neck [2]. The structural organization of the pharyngeal arches involves components from each of the primary endoderm, mesoderm and ectoderm layers. Recent embryological research has highlighted the importance of the endoderm in pharyngeal arch formation, segmentation and its role in tissue specific differentiation. Recently investigated signaling pathways are not fully understood [3].

## Methods

The comprehensive history, diagnosis, radiology details and open surgery operative details were prospectively kept in this rare cohort of patients. The same consultant surgeon treated and operated on all four cases and continued their long-term follow up at the Royal National Throat, Nose and Ear Hospital, London, UK.

## Results

Four cases of 3rd or 4th pharyngeal arch anomalies were followed-up and treated. Three of these patients were male, and one female. Three cases had become initially symptomatic in childhood and all of them presented as acute left sided neck infections, without signs of a sinus or fistula. Further recurrent infections were also left sided. Fistulas only developed after spontaneous rupture of the neck abscess and incision and drainage procedures. Three of these patients had considerable life changing, long-term morbidity.

### Case 1

This female patient did not primarily present until 24 years of age. She had an acute painful, left neck swelling which was diagnosed as viral thyroiditis. She was treated with steroids and antibiotics for 1 month. Her symptoms and left neck swelling recurred after 14 months and she underwent left thyroid lobectomy. Only 1 month later, she required incision and drainage of a new left neck abscess. Between 28 and 34 years of age she had three further left neck abscesses requiring incision and drainage. At 35 years of age, she re-presented with a new acute left neck swelling. MRI, thyroid scans and pharyngoscopy did not demonstrate an internal piriform sinus opening. At left neck exploration, a mass of fibrous tissue, fascia, scarred left sternothyroid muscle and residual tract was excised. The tract was demonstrated adjacent to the left cricoid cartilage and crico-thyroid muscle, 3–4 mm anterior to the inferior thyroid cornu and left recurrent laryngeal nerve. All involved tissue was removed with additional operating microscope control. At 25-year follow-up she had no further recurrence of her previous extensive problems.

### Case 2

This male patient initially presented at 4 years of age with a left neck swelling which was treated with antibiotics with several further episodes between the ages of 5 and 8 years, at which point he developed a discharging left neck sinus. Ongoing infections with multiple operations caused severe disruption to his education. At 25 years of age, investigations including microbiology, CT, examination under anesthesia and pharyngoscopy did not show an internal piriform sinus opening. At left neck exploration substantial amounts of peri thyroid scar tissue and residual left thyroid lobe were excised. A further abscess drainage was undertaken 3 months later. Between 25 and 33 years of age the patient had intermittent pus draining from a left mid-neck sinus with recurrent infection. During this period he also had a left tonsillectomy and neck exploration with no subsequent improvement.

At age 33 a pharyngoscopy did not demonstrate an internal opening from the piriform fossa. The neck was again re-explored and all gross scar tissue and involved fascia was excised medial to the deep aspect of sternomastoid muscle, around the carotid sheath and along the posterior border of the left thyroid cartilage. There was an obvious tract attached to the inferior thyroid cartilage border adjacent to the inferior cornu and left recurrent laryngeal nerve. All tract and involved periosteuum and a small portion of the cricoid and thyroid cartilage were removed, under operating microscope control, to ensure that all abnormal tissue was excised. The left sternomastoid muscle was released inferiorly and transposed into the excised area to aid healing. The patient remained well without recurrent symptoms for 26 years but experienced a single episode of neck swelling in 2018, which settled with antibiotics.

### Case 3

**Fig. 1:**
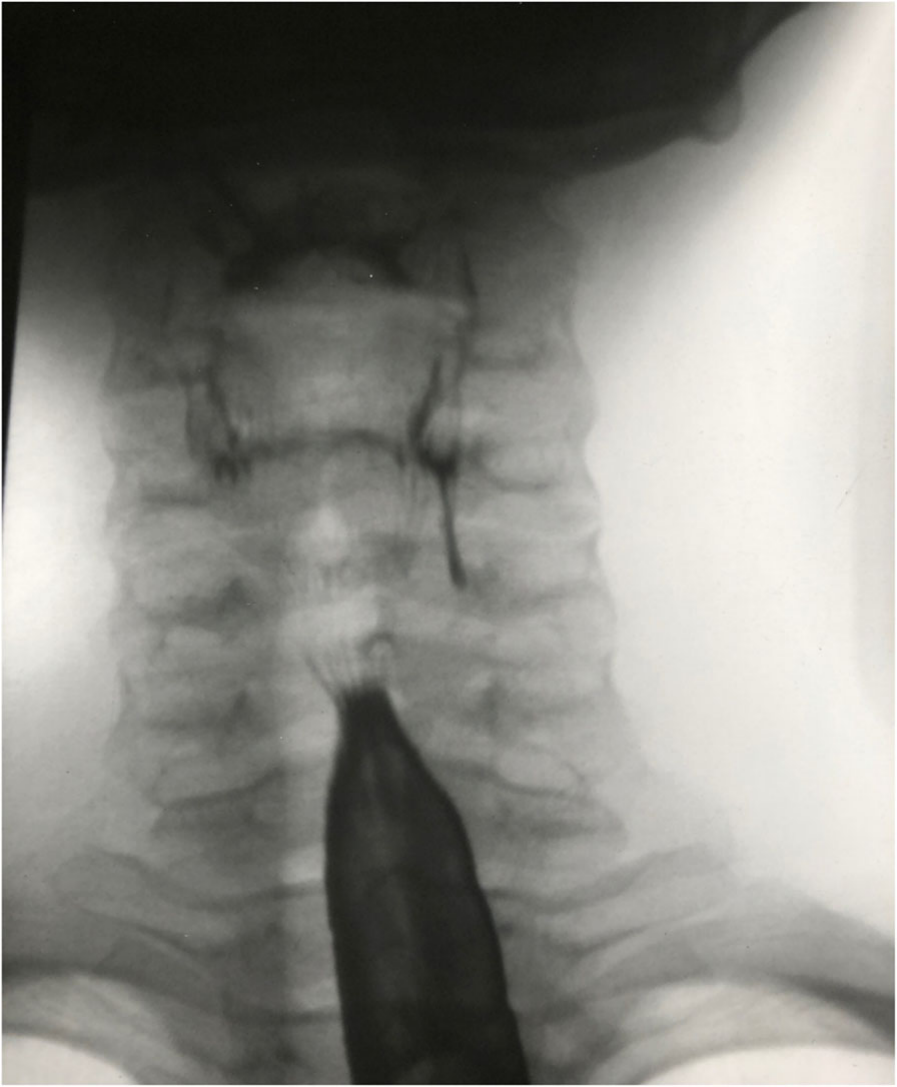
Barium swallow with rapid sequencing images showing a 4 cm. blind-ended sinus from the apex of the left piriform fossa extending inferiorly in relation to the deep aspect of the lobe of the thyroid. Additional imaging was undertaken with ultrasound

This teenage boy presented at 14 years of age, having had two episodes of left neck pain and swelling. He was initially diagnosed as having viral thyroiditis. Three weeks later, there was a sudden deterioration with formation of a large bilateral neck abscess, maximal on the left side. This was incised and drained and he spent 6 days as an inpatient. Subsequent ultrasound and barium swallow showed a 4 cm long sinus tract extending postero-medially to the left lobe of the thyroid gland demonstrated in Fig. 1. Direct pharyngoscopy showed an opening at the apex at the left piriform fossa. He underwent left neck exploration, at which the whole sinus tract was excised en bloc, including a partial left thyroid lobectomy and all associated involved carotid fascia and sternothyroid muscle. The sinus tract extended from the piriform fossa to within a few milimetres of the left recurrent laryngeal nerve at the cricothyroid joint. Now, 35 years old the patient remains well after 21-year follow-up without further recurrence.

### Case 4

This patient first presented at 9 years of age, with a left neck abscess which required incision and drainage. Twenty-one years later, at the age of 30, he had a new left neck swelling which settled with antibiotics. Aged 39 he presented with a large left neck abscess which required incision, drainage and antibiotics. Pharyngoscopy 1 year later did not reveal any piriform fossa internal opening. At elective left neck exploration, all scar tissue, abscess cavity remnant, involved left sternothyroid and inferior constrictor muscle adjacent to the left piriform fossa mucosa was excised. Thyroid lobectomy was not required. The patient remains well after 27-year follow-up.

## Discussion

Long-term follow-up, of more than two decades, has shown probable cure of all the patients following open surgery. Their presentations are in line with the published literature, where more than 95% are left sided, almost 100% present with neck infection [1]. Sinus and fistula formation to the skin seem to only occur after spontaneous rupture of an abscess or after incision and drainage operative procedures [1]. Initial presentation occurs in both children and adults. These four cases emphasize the extent of the time taken between the initial presentation, the diagnosis and definitive treatment leading to considerable morbidity.

Internal openings in the apex of the piriform fossa may be clearly defined, as presented in Case 3, but after multiple infections and surgery, are not always found. Surgeons continue to explore all possible ways to carry out procedures endoscopically with consequent reduction in morbidity and inpatient stay. Lachance & Chadha (2015), carried out a systematic review of endoscopic obliteration techniques in children and concluded that it was successful in 90% of primary cases and 87% of revision cases [4]. It is understandable that endoscopic procedures should be preferable to open surgery in childhood but the follow up times in all recent papers are relatively short and long-term reports are not available. As is demonstrated in our cases, recurrence can occur over decades, well into adulthood. Whilst the initial part of any piriform sinus or fistula tract might be obliterated endoscopically, the lengthy and scarred tracts found in three of the patients described herein could not have been accessed from the pharynx and obliterated by any endoscopic procedure.

The pathology of the excised tissue in these patients had notable epithelial remnants, thyroid tissue, chronic inflammation and scarring. Cases 1, 2 and 3 showed definite tracts ending at the lower border of the thyroid cartilage and adjacent to the cricothyroid muscle, closely related to the laryngeal point of entry of the recurrent laryngeal nerve. Embryology textbooks describe pharyngeal arch development in humans occurring as a series of paired arches in the embryo [5]. Every arch has its own nerve that controls a distinct muscle group, artery, and skeletal tissue. They grow and fuse ventrally in the midline. Each arch is surrounded by mesenchyme and the arch development is staggered and still controversial. Accounts vary in publications and the precise aetiology of the 3rd and 4th arch pharyngeal anomalies remain unclear.

An alternative embryological explanation for these clinical cases has been proposed, suggesting that they are persisting remnants of the thymopharyngeal duct [1]. This duct which forms as the thymus, descends from the ventral portion of the third pouch through the fourth arch and eventually fuses with the contralateral thymus. Failure of this duct to close would leave a sinus arising from the piriform fossa. The thymus descends during the 7th and 8th weeks at the same time as the thyroid and the involvement of the left thyroid lobe, to a greater or lesser degree in these cases, and is in keeping with this embryology. The clinical aspect of these recurrent cases has a marked similarity to the presentation and problems due to inadequate excision of the entire thyroglossal tract, in the much more common congenital anomalies of the thyroglossal duct [6]. Thymopharyngeal duct cysts have been described in the literature in an identical position and distribution to our cases [7].

Whilst no patients have been reported in the literature showing the theoretical extensive classical path of a complete 4th pharyngeal arch anomaly, rare cases such that of Godin et al. [8], have shown sinus tracts extending inferiorly as far as the clavicle. The problem with postulating the exact embryological pathway is complicated by the fact that many cases are associated with extensive infection and abscess formation, as demonstrated. Sinuses and fistulae can also be created by recurrent infections and surgical procedures. It is therefore essential in the cases with recurrent multiple infections that the entire area which has been involved in any inflammatory mass is meticulously excised at open surgery, with removal of the left lobe of the thyroid gland, fascia and any involved strap muscles. The overall surgical approach should be philosophically comparable to a selective neck dissection rather than a localized excision. The inferior constrictor muscle must be divided sufficiently to give excellent exposure to allow high ligation of the tract at any piriform fossa internal opening. Additional careful dissection inferiorly to the inferior cornu of the thyroid cartilage and cricoid cartilage, with preservation of the recurrent laryngeal nerve may be necessary, as in our cases, with use of loupes or the operating microscope aiding this dissection. Healthy sterno-mastoid muscle can be detached from its inferior insertion, mobilised and transposed antero-medially, (based on its superior segmental blood supply) into the defect to aid healing.

The inferior constrictor muscle must be divided to allow high ligation of the tract at any piriform fossa internal opening. Additional careful dissection inferiorly to the inferior cornu of the thyroid cartilage and cricoid cartilage, with preservation of the recurrent laryngeal nerve, may be necessary, as described with use of the operating microscope aiding this dissection. Healthy sterno-mastoid muscle can be transposed into the defect to aid healing.

The main strengths of this case-series is the length of the follow-up, confirming success in surgical management of this condition. Although, it provides the supplemental opinion to embryological developments of the third and fourth branch anomalies, the detailed surgical technique has not been discussed here. The rarity of the cases and the uncertainty of embryological development of these arches, makes this report not a definitive or conclusive word on the matter. Moreover, the literature describing the long-term follow-up following the endoscopic treatment of these anomalies is not available as of yet. However, it would be important to address the comparatives of these different techniques in the future.

## Conclusion

In cases of multiple and recurrent neck abscesses a diagnosis of 3rd or 4th arch anomaly should be considered. To achieve definitive management these cases require detailed dissection from the piriform fossa infero-laterally to include all involved tissue adjacent to the carotid sheath, left thyroid lobe, thyroid and cricoid cartilages with preservation of a recurrent laryngeal nerve.

Modern embryological research suggests that development of the pharyngeal apparatus is a truly complex process that relies on genetic signaling from the endoderm, ectoderm and mesoderm that control proliferation, migration and morphology. A full understanding of these pathways requires more work to elucidate the many congenital anomalies of the pharyngeal apparatus [9]. Recent embryological research emphasises the importance of the endoderm in human development [9], with alterations in third pouch development that should be considered.

## Data Availability

Data sharing is not applicable to this article as no datasets were generated or analysed during the current study.

## Abbreviation

CT: Computed tomography
